# Antenatal testing for anaemia, HIV and syphilis in Indonesia – a health systems analysis of low coverage

**DOI:** 10.1186/s12884-020-02993-x

**Published:** 2020-05-29

**Authors:** C. Baker, R. Limato, P. Tumbelaka, B. B. Rewari, S. Nasir, R. Ahmed, M. Taegtmeyer

**Affiliations:** 1 Liverpool School of Tropical Medicine, Pembroke Place, Liverpool, L3 5QA UK; 2 Eijkman Institute for Molecular Biology, Jl. Diponegoro No. 69, Jakarta, 10430 Indonesia; 3 Division of Communicable Disease, World Health Organization Regional Office for the Western Pacific, Manila, Philippines; 4 Faculty of Public Health, Universitas Hasanuddin, Makassar, Indonesia

**Keywords:** Indonesia, Antenatal testing, HIV, Syphilis, Anaemia, Decentralisation

## Abstract

**Background:**

Adverse pregnancy outcomes can be prevented through the early detection and treatment of anaemia, HIV and syphilis during the antenatal period. Rates of testing for anaemia, HIV and syphilis among women attending antenatal services in Indonesia are low, despite its mandate in national guidelines and international policy.

**Methods:**

Midwife-held antenatal care records for 2015 from 8 villages in 2 sub-districts within Cianjur district were reviewed, alongside the available sub-district *Puskesmas* (Community Health Centre) maternity and laboratory records. We conducted four focus group discussions with *kaders* (community health workers) (*n* = 16) and midwives (*n* = 9), and 13 semi-structured interviews with laboratory and counselling, public sector maternity and HIV management and relevant non-governmental organisation staff. Participants were recruited from village, sub-district, district and national level as relevant to role.

**Results:**

We were unable to find a single recorded result of antenatal testing for HIV, syphilis or anaemia in the village (566 women) or *Puskesmas* records (2816 women) for 2015. Laboratory records did not specifically identify antenatal women. Participants described conducting and reporting testing in a largely ad hoc manner; relying on referral to health facilities based on clinical suspicion or separate non-maternity voluntary counselling and testing programs. Participants recognized significant systematic challenges with key differences between the more acceptable (and reportedly more often implemented) haemoglobin testing and the less acceptable (and barely implemented) HIV and syphilis testing. However, a clear need for leadership and accountability emerged as an important factor for prioritizing antenatal testing and addressing these testing gaps.

**Conclusions:**

Practical solutions such as revised registers, availability of point-of-care tests and capacity building of field staff will therefore need to be accompanied by both funding and political will to coordinate, prioritize and be accountable for testing in pregnancy.

## Background

Tests for anaemia, HIV and syphilis, all leading preventable causes of adverse pregnancy outcomes, are an essential component of antenatal care (ANC) [1]. Anaemia in pregnancy is a significant public health problem in Indonesia with a prevalence of 37% [2]. It increases the risk of prematurity and low birthweight, and contributes to mortality from obstetric haemorrhage, which is estimated to cause 25% of maternal deaths in Indonesia [3, 4]. There is evidence increasing numbers of women of reproductive age in Indonesia are HIV positive [5]. Untreated HIV and syphilis in pregnancy are transmitted to the infant in around 30 and 15% of cases respectively [6, 7]. Untreated syphilis in pregnancy is also associated with an increased risk of perinatal death, prematurity and low birthweight [7]. Appropriate treatment of anaemia, HIV and syphilis during pregnancy significantly reduces these risks of adverse outcomes for mother and baby. All three are therefore key priorities in global health, exemplified in campaigns to end preventable maternal mortality and eliminate mother-to-child transmission of HIV and syphilis [8, 9]. Similarly, Indonesian health policy stipulates that all pregnant women should receive haemoglobin, HIV and syphilis tests in the first trimester [10, 11]. The Indonesian government has also committed to the elimination of mother-to-child transmission of HIV and syphilis by 2020 [12]; mandating at least 95% coverage of HIV and syphilis testing among pregnant women [9]. Since 1989, Indonesia’s Village Midwife Program has led to increased midwife numbers and density [13] and improved first trimester ANC attendance [14]. Despite this context of national and international guidance and 87.8% of pregnant women attending at least four ANC visits [15], available evidence from Indonesia indicates suboptimal levels of testing for anaemia, HIV and syphilis. Only 41% of pregnant women report receiving any blood tests [15]. 2015 HIV and syphilis testing rates were < 2%, although HIV testing coverage rose to 28% in 2017 [16, 17] .

Maternal health was prioritised through decentralisation of the Indonesian health system in 2001. Budgetary and service delivery responsibilities were devolved to provincial and district governments, each of which has a health office [18, 19] (Table 1). However, the MoH can bypass decentralisation to fund specific programmes, such as in maternal and child health. Relationships between health offices are not hierarchical, with DHO and PHO accountable to their specific level of government rather than to the PHO or Ministry of Health (MoH) [19]. ANC guidelines state they are flexible to allow adaptation of guidelines by DHOs [20]. In this context, minimum service standards (MSS) were developed to help ensure minimum standards of care delivery, but are described as complex and not universally adhered to [21, 22].

**Table 1:** Summary of key roles and responsibilities in Indonesia’s decentralised health system

	Key roles	Accountable to
**Ministry of Health (MoH)**	Technical support and guidelines for P/DHO Regulation Management of personnel and social insurance programmes Operation of some tertiary hospitals	National government
**Provincial Health Office (PHO)**	Coordination of healthcare in province Management of provincial health budget Operation of provincial hospitals	Provincial government
**District Health Office (DHO)**	Management of district health budget Adaptation of guidelines Operation of district hospitals Operation of primary health care through the Puskesmas* and associated services	District government

Detail from Indonesia health system review [19].

*Sub-district health centre

ANC is accessed via the midwife-led clinic in sub-district health centres (known as the *Puskesmas)* or through the midwife-led *Posyandu,* a community level outreach clinic. These are within the authority of the DHO, but each *Puskesmas* has its own Head, responsible for its services. The monthly *Posyandu* is arranged by *kaders* (community health volunteers), who play an interface role between the communities and the formal health sector; identifying and registering pregnant women and supporting midwives. *Posyandu* services are described in the ‘10 T’ and include maternal weight; fundal height, foetal position and heartbeat; tetanus immunisation; iron supplementation, pregnancy counselling and laboratory testing (haemoglobin, HIV, syphilis and urinary protein) [23]. In practice the 10 T package is variably implemented across and within districts [24].

Addressing the gap between (inter) national guidelines and the current decentralised approach to antenatal care requires health system solutions that support testing at *Posyandu* and *Puskesmas* level. Anaemia, HIV and syphilis can all be screened for with simple rapid diagnostic tests at the point-of-care, making them feasible to implement at the *Posyandu* [25–28]*.* Indonesian guidelines offer these as an option for increasing coverage [10, 23]. We set out to document and explore current practice as well as barriers and enablers in the delivery of antenatal testing for anaemia, HIV and syphilis in the context of the decentralised health system.

## Methods

### Study site

The study was conducted in 2016 in Cianjur district, West Java province, where the study team had an existing relationship with maternity services through the REACHOUT consortium [29]. Cianjur is predominantly Muslim and has 2.2 million people. The main town, Cianjur, has 165,000 residents, but much of the district is rural, with non-tarmac roads between villages. 40% of district residents work in agriculture, particularly tea and rice production, and 20–25% in retail and hospitality [30]. Cianjur has 32 sub-districts (with populations of 60–90,000), 45 *Puskesmas* and 2846 *Posyandu* [31]. In West Java as a whole, any ANC attendance is 96.2 and 63.3% of deliveries occur in health facility [15]. Antenatal HIV prevalence data are not available for West Java but HIV prevalence is 8.3% among female sex workers and 12.8% among injecting drug users [32].

### Study design

We used mixed methods to provide a more complete description of current practice [33]. After conducting a routine record review to understand the extent of antenatal testing, we used qualitative methods to explore perceptions and experiences of maternal health stakeholders and explain the testing rates observed in the records [34]. We used focus group discussions (FGDs) to generate discussion and explore shared understandings of antenatal testing of those with the same job role, minimising power differentials and increasing confidence and engagement in discussing topics such as HIV [35]. We used semi-structured interviews (SSI) at sub-district, district and national level to gain insights from the unique perspectives of selected informants with a variety of roles in antenatal testing [36].

### Record observation

Eight villages (four in Ciranjang sub-district and four in Gekbrong sub-district) at varying distance from the *Puskesmas* (defined as greater or less than 5 km) were selected for record review. Midwife-held logbooks for all pregnant women attending ANC for the year 2015 were retrospectively reviewed for data on anaemia, HIV and syphilis testing and compared to ‘Integrated ANC Reports’ and laboratory records at the respective *Puskesmas,* and to an electronic copy of the District Heath Office (DHO) maternal health report.

### Qualitative data

#### Participant selection and recruitment

Qualitative interviews were held in Ciranjang sub-district. Using the same four villages as for record review, we purposively sampled participants with maternal and child health (MCH) roles and included non-governmental organisations (NGOs) providing HIV services to create a divergent sample representative of the health system [37]. In addition, snowball sampling identified additional key informants from the *Puskesmas*, district and national levels [37] (see Table 2). Participants received written information sheets in Bahasa (translated by research team), or in English at national level, and were given opportunity to reflect and ask additional questions before written informed consent was taken.

**Table 2:** Participant characteristics, data mode, location and level

Job area	Data mode	Participants	Years of participant experience in role (mean)	Location	Level
Kader	FGD	8	15.4	Karangwangi	Village
	FGD	8		Ciranjang	Village
Midwife	FGD	3	8.1	*Puskesmas* Ciranjang	Sub-district
	FGD	6		Mekargalih, Ciranjang, Sindangsari and Karangwangi	Village
**Total**	**FGD (4)**	**25**			
Laboratory testing and counselling	SSI (3)	3	10.4	*Puskesmas* Ciranjang	Sub-district
	SSI	1		Cianjur District Health Office	District
	SSI	1		Ministry of Health	National
Public sector MCH & HIV management	SSI (2)	2	8.0	*Puskesmas* Ciranjang	Sub-district
	SSI (2)	2		Cianjur District Health Office	District
	SSI	3		Ministry of Health	National
NGOs	SSI (3)	3	5.3	NGO National Offices	National
**Total**	**SSI (13)**	**15**			

#### Data collection and analysis

Similar topic guides were used for the FGDs with *kaders* and midwives as for the semi-structured interviews with key informants, with adjustments to suit roles and expertise. Questions focused on the purpose and policy of antenatal testing, current practice, and perceptions of implementing testing, progressing from less to more sensitive or difficult topics [36], typically anaemia, followed by HIV and lastly, syphilis. An example topic guide may be found in the supplementary materials that accompany this article. Tools were amended after a pilot FGD [38]. FGDs lasted 60–90 min. Data at district, sub-district and village levels were collected by experienced qualitative researchers in Sundanese and/or Bahasa. National level data were collected by the first author in English. Daily team debriefs were conducted to probe emerging themes and noted in a research diary. Interviews were digitally recorded, transcribed and translated into English, checked by a bilingual researcher and sections that lacked clarity were discussed further. Transcripts were analysed in English using an inductive framework approach [39] with the support of NVivo 10 software. Themes were identified from contrasts and comparisons across participant role, administrative level and condition (anaemia, HIV or syphilis) using charts constructed from coding. Refutational searches were carried out to ensure contradictory opinions and differences across roles and administrative levels were detected.

### Ethical considerations

The Liverpool School of Tropical Medicine Master Review Panel 2016 [9] and the Research and Ethical Committee of University Hassanudin (UH 14120659) approved the study. Permission and access to antenatal records was granted by Badan Kesatuan Bagsa Dan Politik Kabupaten, Cianjur (District Health Office).

## Results

We retrospectively reviewed village records for 566 pregnant women and *Puskesmas* level records for 2816 first ANC attendances in Ciranjang (*n* = 1699) and Gekbrong (*n* = 1117) covering the period January to December 2015. There were no records of testing within 2015 ANC records. The pre-2010 version of the midwife-held logbook and *Puskesmas* Integrated ANC record was used in all study sites. This was not designed for recording universal testing; there was a field to record severe anaemia (but no ticks found against 566 entries) and no fields for recording HIV or syphilis testing (Table 3). One midwife shared that she had manually added a column for haemoglobin result to her 2016 logbook because she wanted to record the results of free haemoglobin tests offered by a new programme in her area. Only 24.6% of pregnant women in her record in 2016 had a haemoglobin result recorded. The DHO MCH report was the only maternity record reviewed which contained fields to record haemoglobin, HIV and syphilis testing results, but all were completed as “data not available”.

**Table 3:** Antenatal testing data fields in medical facility records in Puskesmas Ciranjang and Cianjur district reports

Record	Data fields relevant to antenatal testing			
	Patient	Haemoglobin (Hb)	HIV	Syphilis
*Posyandu* register	Individual identifiers	None	None	None
Midwife-held logbook of pregnant women	Individual identifiers	Tick if Hb < 8 g/dL Column manually added for Hb value (g/dL) in one village in 2016	None	None
*Puskesmas* integrated ANC report	No individual identifiers Individuals separated and grouped by village	Copied from midwife records Tick if Hb < 10 g/dL	None	None
*Puskesmas* laboratory Full Blood Count record	Gender, age, geographical origin	Hb value (g/dL)	None	None
*Puskesmas* laboratory HIV and syphilis record	Patient code, no other identifiers	None	Positive/negative	Positive/negative
DHO MCH report	No individual identifiers Aggregated data: ANC attendance by sub-district; testing data by district	Number pregnant women tested Number with anaemia (8-11 g/dL) and (< 8 g/dL)	Number offered test and number tested Number positive and number treated	Number checked for STI* Number with STI and number treated

*STI data not broken down by type

It was not possible to link *Puskesmas* laboratory records with ANC records to determine testing rates due to a lack of patient identifiers in laboratory records (Table 3). During 2015, the *Puskesmas* Ciranjang laboratory performed a total of 90 HIV and 23 syphilis tests among the general population, although no data was available on how many were pregnant women. *Puskesmas* Gekbrong did not have a laboratory, and HIV and syphilis tests were conducted by the DHO. There were three cases of HIV recorded in the same year, but none among pregnant women.

### Qualitative results

Very low rates of antenatal testing coverage were described by participants at all levels who reflected on their experiences of (in)effective implementation of national policy at district level. Coverage of haemoglobin testing was described as higher than HIV or syphilis testing, but largely reliant on referral of at-risk patients to the *Puskesmas* laboratory. Emerging themes grouped easily into the six building blocks outlined in the World Health Organisation’s Health Systems Framework [40]. Barriers to implementation are summarised in Table 4 and varied by the type of test that was being discussed. The findings from *kaders* are reflected in discussions about service delivery as this is where they interface with the health sector most commonly. Key findings for each theme reflect commonly held views across the range of participants at national, district and community levels, except where views restricted by participant type or individual are specified.

**Table 4:** Perceptions of health system barriers to antenatal testing

Health System Block	Haemoglobin testing	HIV and syphilis testing
Leadership and governance	Some level of policy awareness. Limited accountability for process indicators. Regarded as midwife-led service but no descriptions of supportive supervision of midwives by non-national participants.	National policy on universal testing not widely disseminated - poor understanding at village level. Testing for HIV and syphilis not seen as a priority intervention with no reported inclusion in district antenatal strategy. No indicators set; no reported follow-up or feedback on available data.
Health care financing	Sahli method chosen* as cheaper but some concerns raised about quality. Insufficient funding for free testing seen as limiting.	Multiple small-scale funding sources, often from donors for pilot programmes. Rapid diagnostic tests seen as expensive. Cost effectiveness not discussed by participants.
Health workforce	Many report midwives have limited practical experience with Sahli method. Midwives report feeling too busy to conduct testing or complete 10 T**.	Midwives and *kaders* aware of risk of vertical transmission but little knowledge on effectiveness of prevention. Midwives lack training on rapid diagnostic testing for HIV and syphilis and describe being ‘afraid’ to do counselling. Laboratory staff aware of algorithms but focused on high risk patients due to limited resources, and perceive testing as their role, concerned that quality cannot be maintained if task-shifted outside laboratory. All levels perceive a need for counselling training as a system barrier. Shortages of laboratory personnel and counsellors described, but only national informants discussed future possibility of task-shifting to midwives.
Medical products and technologies	Sahli accuracy seen as acceptable by all if midwives skilled. Managers concerned about other rapid haemoglobin tests, some of which were favoured by midwives as easier. Supplies of Sahli kit unreliable.	Reported reliance on laboratory method (RPR for syphilis; lab ELISAs for HIV) and quality assurance systems. Little awareness of syphilis RDT at sub-national levels. Key informants report delays in procurement of lab-based tests due to communication between different administrative levels and departments.
Information and research	Large amount of missing data on haemoglobin testing in ANC records not recognised as a problem.	Difficulties reported with integration of HIV testing into ANC data – no data fields, no reporting systems, no tracking. Difficulties reported with integration of syphilis testing with ANC data – no data fields, no reporting systems, no tracking.
Service Delivery	Low coverage seen as suboptimal but feasible to increase within current system. Limited community demand for service delivery. Reliance on referral to *Puskesmas* on clinical suspicion but community participants describe low uptake due to cost, time and fear.	Testing all done at *Puskesmas* or district level. Recognition there is very little HIV or syphilis testing happening. National informants aware of integrated ANC testing pilot sites. No demand for testing described with perception of low prevalence. Stigma a significant barrier to testing amongst providers and community. Referral only on clinical suspicion – not opt out or routine testing delivery model. Relies on referral to *Puskesmas* and community participants describe low uptake due to cost, time and fear.

* Suggested rapid tests outlined in national Regulation 25. District staff made financial decision to choose this method

** ‘10 T’ a package of ten expected steps for examination, testing and treatment of pregnant women at antenatal care

### Leadership and governance of antenatal testing

We found a significant degree of ‘policy evaporation’ with low levels of knowledge and implementation of national ANC testing policies. Participants at all levels knew haemoglobin testing to be universal, but *kaders* were unclear on the frequency required. Very few participants (6 national and 1 *Puskesmas* key informant) realised HIV and syphilis testing were routinely recommended or were able to articulate the benefits of testing in early pregnancy. Many thought policy recommended testing pregnant women considered ‘at risk’, as illustrated in this typical quote:

*“Our aim is not the general screening but screening based on indications. So, we aimed the pregnant women with tattoo, pregnant women whose husband work as driver, pregnant women who complain about leucorrhoea [vaginal discharge].”* (SSI, *Puskesmas*).

Low testing coverage of HIV and syphilis was not seen as a problem at any level of the system, nor was addressing coverage a priority due to perceived low prevalence rates among pregnant women. While high maternal mortality rates in Cianjur were raised by district key informants as a concern, only one mentioned the link between mortality and antenatal testing rates. The lack of indicators, reporting, feedback, follow-up and accountability at all levels left little incentive or motivation to address low coverage. A national participant linked this to decentralization:

*“When you do haemoglobin testing and report it back, you know the proportions of pregnant women who are anaemic, and then you use that for planning you can reduce the cases because you collect the data. Central level have the policy, but implementation is another challenge because of decentralisation.”* (SSI, NGO National).

Targets and indicators under local leadership were seen as a motivator for effective programming by the key informants at national and district level. A few talked about improving local accountability through involving professional associations in making testing routine. Most participants felt it came down to vertically imposing tasks and targets, as this participant summarized:

*“If the head of district cares about this [HIV and syphilis testing], he can control the head of district health office to make this work and it can be seen from the health indicator.”* (SSI, *Puskesmas*).

### Health care financing and priority-setting for antenatal testing

Co-funding (national and district) for antenatal testing was described as the responsibility of the district level decision-makers and competing priorities meant that measuring and improving antenatal testing coverage came lower down their funding list, as shown in this quote:

*“The fact is the dynamic in every district and the priority of every district is different”* (SSI, NGO, National).

Many key informants described low cost as the rationale for implementing the Sahli method, although some expressed concerns regarding its quality. Some also reported available funds could not support free universal haemoglobin testing or required staff training, thus limiting the service. Several informants described fund allocations for HIV and syphilis testing coming from non-district sources (international donors, pilot programmes) with impacts on coordination, equipment, supply chains, disbursement and sustainability, as illustrated by this district-level key informant.

*“In the past, we also have the fund to do that [universal HIV screening], but now they limit the fund [ie funding reduced or terminated].”* (SSI, *Puskesmas).*

Rapid diagnostic tests for HIV and syphilis and the accompanying training needs were seen as expensive. None of the participants, however, discussed the cost effectiveness of identifying and treating cases, preventing illness and transmission.

### Health workforce

There was a common perception that testing was the remit of laboratory staff and should remain centralised to ensure quality, although key informants pointed out significant shortages of laboratory staff. All midwives mentioned *Posyandu* workload as a barrier to haemoglobin testing.

*“In Posyandu we have to do everything by ourselves, like immunisation … we are very busy... Sometime the pregnant women want to get home early and if we hold them little longer they don’t want to come to Posyandu again”* (FGD, Midwife).

All informants, including midwives themselves, mentioned that midwives’ skills in performing the Sahli test were sub-optimal. Very little mention was made of supervision happening, although a few national informants raised it as a potential way of following-up issues around capacity and reporting.

*“The role of the district office is to perform this supportive supervision to midwives. … So there’s no reason [not to do the haemoglobin test].”* (SSI, Public Sector National).

Most informants identified a lack of training and skills. Counselling was seen as necessary to conducting HIV or syphilis testing, but specialized, thus posing a major barrier.

*“Yes, those [HIV and syphilis] tests are available, but we are afraid about the suggestions to the patients, because it relates to the counsellor.”* (FGD, *Puskesmas* Midwife).

A few key informants raised linked concerns about accreditation of testers, and whether and how this would be provided in the current setting. They expressed multiple layers of collaboration, complications and bureaucracy that would need to be overcome before rapid testing policies could be put into practice. The central lab, district laboratories, Family Health Directorate and Human Resources Directorate were all identified as significant stakeholders.

### Potential of medical products and technologies

Overall, we found little mention of the potential for increasing point-of-care haemoglobin testing or adopting new technologies for HIV and syphilis testing during the *Posyandu*. Instead, participants discussed the time it took to perform a Sahli test (with one midwife calling for a simpler to use kit) and the supply of Sahli testing kits. Most participants had no knowledge of, or experience with, rapid HIV and syphilis testing and the relative advantages of new technologies. Discussion of ease-of-use and accuracy issues for newer technologies was not found in our data.

### Information and research on antenatal testing almost completely absent

Our qualitative findings confirmed the findings from register reviews that data collection, analysis and use was all but absent at district level. Data for haemoglobin testing were felt to be incomplete, unlinked and poorly aggregated. Data on HIV and syphilis testing was not part of routine antenatal care reporting. This lack of integration was also described at national level with separate forms for HIV and maternal health. The need for research and documentation of the knowledge gaps did not arise among district level participants and, when discussed among national key informants, research agendas were described as externally driven, with little local ownership.

### Service delivery gaps recognised but not seen as a concern

There was widespread awareness that low coverage of antenatal testing was the norm, yet this was not raised as a concern by *kaders* and midwives, who perceived that it was not clinically indicated when women appeared healthy. Descriptions of haemoglobin testing being conducted at the *Posyandu* level were rare. Most informants concurred that midwives referred symptomatic patients to the *Puskesmas*.

“… *but if there is no indication, we don’t ask them to get tested, if mothers look normal. If mothers look pale and feel dizzy, we refer them* (FGD, Midwife).

Midwives and *kaders* stated haemoglobin testing was acceptable to women, but they did not take up referrals because of cost, time and limited understanding of the need for testing. All informants in Cianjur agreed that coverage of HIV and syphilis testing was low in pregnant women and, with one exception, all brought up the issue of stigma as significant obstacle to HIV (and syphilis) testing. Stigma was felt to have affected community and provider acceptance of testing with impacts on decision-making and prioritisation at higher levels. A few mentioned that making testing routine could normalize it, reducing stigma.

*“It [HIV and syphilis testing] must be for all people. If only particular people, the community will talk.”* (SSI, District).

## Discussion

Our findings from Cianjur district reveal that the decentralized context and complex interactions between multiple actors led to policy evaporation from national to district and district to community level. Antenatal testing was limited and, when available, neither conducted nor reported in a systematic manner. There was no evidence in *Puskesmas* or *posyandu* ANC records in either sub-district studied that any women were tested for anaemia, HIV or syphilis, suggesting none are likely to have received any indicated treatment for these conditions. Analysis of the six health systems building blocks showed participants describing significant challenges to implementation with key differences between the more acceptable (and more often implemented) haemoglobin testing and the less acceptable (and barely implemented) HIV and syphilis testing. The reliance on referral for testing based on clinical suspicion further played into the stigma dynamics, making referral hard to initiate and take up. Despite major systematic gaps being described and recognized, antenatal testing was not regarded as of sufficient priority in this presumed low prevalence setting. We found that coordination of testing lacked leadership, prioritization and accountability across all levels of the decentralized system.

The decentralization of fiscal and administrative responsibilities has been previously described as affecting service quality and priority setting for health in Indonesia [41, 42]. Changes in accountability, coordination and information flow following decentralisation mean there are no clear lines of responsibility [43], illustrated in our study in relation to low antenatal testing rates. There are also challenges in acceptability, priority setting and health care financing. Haemoglobin testing was poorly implemented despite being widely accepted [44]. For HIV and syphilis, our finding of stigma in community, healthcare and decision-making arenas is consistent with other evidence from Indonesia [45] and other contexts [46]. In the Philippines, stigma has had a negative effect on local prioritization of antenatal HIV and syphilis testing in a decentralized health system [47]. A lack of leadership and of political and fiscal prioritization of antenatal HIV and syphilis testing has provided significant challenges in other low prevalence countries despite strong evidence of health benefit and of cost-effectiveness [48, 49]. A 2015 policy analysis of the low political priority for syphilis testing in China revealed similar findings to ours, with limited accountability, perceived high costs and failure to address stigma [50]. However, the same analysis demonstrated that actions to address these barriers in relation to HIV and communicate the effectiveness of treatment for individuals and society had effectively prioritised and increased antenatal HIV testing rates. Actions included establishing effective information flows, accountability and monitoring, which our study suggests could be facilitated in Indonesia in the first instance by changing to the post-2010 ANC record format designed to support universal testing.

Tension between the drive for accessible services [13] and the restrictive testing policy and practice described by our informants has allowed antenatal testing to fall into a gap between village-level ANC and more centralised, often district-level testing services. The introduction of rapid testing technologies administered at the point-of-care has the potential to bridge this gap. All three tests we included in our study are available as rapid diagnostic tests [51]. Point-of-care tests have been successfully introduced in other settings [28] and have been piloted in the Indonesian village midwife programme. For example, malaria rapid diagnostic testing has been shown to be accurate and compare well to field microscopy when conducted by trained *Posyandu* midwives in Sumba district [52]. The availability of excellent, cost-effective kits is however not enough to ensure accurate and consistent testing. Spreading and sustaining these technologies would need to take into account both the intervention itself and readiness and willingness of the system for introduction [53]. Sub-national informants in our study identified midwife workload as a potential barrier to *Posyandu*-based testing. Similar findings in research on anaemia management in ANC in East Java [2] suggest this may be relevant to other areas across Indonesia. Evidence from other contexts supports the hints in our data that potential obstacles within regulation, personnel roles and training are in politics with laboratory staff in relation to task-shifting, and in ensuring equity even in outreach areas [54–56]. Operational research alongside political prioritisation has been shown to inform and facilitate interventions in staff training and supervision, both identified as needs in our study, leading to improved antenatal testing rates [57]. National-level informants in our study discussed operational pilots of HIV and syphilis testing integrated into ANC, which may explain some of the increase in antenatal HIV testing rates seen between 2015 and 2017, but none of these pilots was underway in an area similar to our study site with predominantly accessible village-level ANC without testing infrastructure.

There were a number of limitations to our study. We were not able to track individual women to understand true ANC testing coverage and or explore their perceptions directly. The qualitative methods we used are not generalizable, although the consistency described above with other studies in the Indonesian health system suggest some findings will be applicable outside Cianjur district. Our methods did not allow assessment of the perceived or actual relative importance of the different factors that emerged as barriers. Triangulation across different informants and methods gives some idea of the perceived importance of different factors, but overall there were small numbers of informants and a focus on only one district in a diverse country. All midwife informants participated in FGDs, which may have introduced pressure to conform in the responses given. Triangulation with midwife semi-structured interviews was not possible with available resources, although differing views within the FGD were noted, such as in relation to understanding of testing policy, details of performing the Sahli test and costs of tests, suggesting midwives still felt able to discuss their views freely.

## Conclusion

This study revealed that antenatal testing policies were not implemented systematically at district level and exposes health system weaknesses as the main reason for this. Among multiple barriers to testing described, a lack of leadership, prioritisation and accountability in the coordination of antenatal testing was identified as an obstacle at all levels of the decentralised health system. Practical solutions such as revised registers, availability of point-of-care tests and capacity building of field staff will therefore need to be accompanied by both funding and political will to coordinate, prioritize and be accountable for testing in pregnancy. The following recommendations arose from our study.

- Create demand for antenatal testing through both raising community awareness and reducing stigma (a bottom-up approach) and increasing political will and district-level leadership alongside widespread test availability (a top-down approach)
- Define roles and responsibilities for antenatal testing at all levels of the system
- Adapt midwife-held and *Puskesmas level* reporting formats and records to include antenatal testing, results and treatment, normalising reporting of testing
- Generate evidence from operational research on the numbers of adverse pregnancy outcomes prevented, cost-effectiveness and efficient delivery models, including linkage to treatment

## Supplementary information

**Additional file 1.** Example topic guide, version for district and national level interviews (English version)

## Data Availability

The data are not publicly available due to them containing information that could compromise research participant privacy and consent. Further information can be sought from the corresponding author [MT].”

## Abbreviations

ANC: Antenatal care
DHO: District Health Office
ELISA: Enzyme-linked immunosorbent assay
FGD: Focus group discussion
Hb: Haemoglobin
HIV: Human immunodeficiency virus
*Kader*: Community health volunteer in Indonesia
MCH: Maternal and child health
MoH: Ministry of health
MSS: Minimum Service Standards
NGO: Non-governmental organisation
*Posyandu*: Midwife-led community outreach clinic in Indonesia
PHO: Provincial health office
*Puskesmas*: Government-mandated community health centres in Indonesia
RPR: Rapid plasma reagin
SSI: Semi-structured interview
STI: Sexually transmitted infection
10 T: Government-mandated antenatal care package in Indonesia
