# Supplementary material for: Antenatal testing for anaemia, HIV and syphilis in Indonesia – a health systems analysis of low coverage
Source: BMC Pregnancy Childbirth. 2020 May 29;20:326. doi: 10.1186/s12884-020-02993-x (PMC7257553; doi:10.1186/s12884-020-02993-x)
Supplement: Supplementary file 1 — Additional file 1. Example topic guide, version for district and national level interviews (English version) [file 12884_2020_2993_MOESM1_ESM.docx]

**District and National SSI**

Background characteristics:

Participant IDs

Interviewer ID

Date

Start time

End time

Section 1. General Information

- 1. What is your job role?
  2. What does this position involve?
  3. How long have been working in this role?

Section 2. Perspectives on testing

2.1 Could you please tell me your views on haemoglobin testing in pregnancy?

2.2 Could you please tell me your views on HIV testing in pregnancy?

2.3 Could you please tell me your views on syphilis testing in pregnancy?

Section 3. The testing system

3.1 Could you summarise the current system of antenatal testing for us? (who is tested, where & method)

3.2 From your observation, what are the main challenges or difficulties facing this system?

3.3 Do you think expanding testing is seen as important? Why/why not?

3.4 If not mentioned, ask if they know what the international guidelines are for testing? (they advise testing every pregnant women for all 3 conditions. Currently, this is not possible in the system in Cianjur and we would like their opinions on ways of testing pregnant women in Cianjur)

3.5 What do you think would be the best way to test for haemoglobin, syphilis and HIV in pregnancy in Cianjur?

Section 4. Point-of-care tests (POCT)

4.1 Could you tell me about point-of-care or rapid diagnostic tests, and their advantages or disadvantages?

4.2 Could you tell us about any programs you know of using HIV, syphilis or haemoglobin POCT in Indonesia? (why used, or why not, successes or not)

4.3 Do you think they would be useful in *posyandu* antenatal care in Cianjur or other rural areas in Indonesia? Why or why not?

4.4 What things would you consider or do if you were going to introduce POCT into *posyandu* antenatal care in Cianjur?

Thank you for talking to us. Is there anything else you would like to say?
